# Penicillins’ Solubility in Supercritical Carbon Dioxide: Modeling by Cubic Equations of States Revisited

**DOI:** 10.3390/antibiotics10121448

**Published:** 2021-11-25

**Authors:** Loubna Nasri

**Affiliations:** 1 Department of Pharmaceutical Engineering, Faculty of Process Engineering, University Constantine 3-Salah Boubnider, Constantine 25000, Algeria; loubna.nasri@univ-constantine3.dz or loubnanasri@yahoo.com; 2 Laboratoire ReMeDD, University Constantine 3-Salah Boubnider, Constantine 25000, Algeria

**Keywords:** supercritical CO_2_, *Penicillin G*, *Penicillin V*, solubility modeling, sublimation pressure, equations of state

## Abstract

Development of processes using green solvents as supercritical fluids (SCFs) depends on the accuracy of modeling and predicting phase equilibrium which is of considerable importance to exploit the use of SCF process at the level of pharmaceutical industries. Solid-Fluid equilibrium modeling is associated to many drawbacks when compressed gas-based models as cubic equations of states (cEoSs) are used. The unavailability of experimental values of solute’s sublimation pressure presents one of the major obstacles to the solubility modeling with this type of models, and thus, its estimation is essential and inevitable. This work is an attempt to address a question regarding “accurate estimated value” of sublimation pressure of two antibiotics *Penicillin G* (benzyl penicillin) and *Penicillin V* (phenoxymethyl penicillin). Toward that, first, cEoSs are provided as the thermodynamics modeling framework and fundamental approach. Second, a discussion and a review of some literature results are given. Third, results are invoked to present a criticism analysis that comes from the use of modified form of Peng-Robinson (PR) equation of states. Finally, considerable improvement of modeling results by using a new sublimation pressure is shown.

## 1. Introduction

Technologies based on SCFs, for which different applications are developed constantly for foods, dyes, polymers and pharmaceuticals processing, are attracting great interest because they use nontoxic and green solvent as carbon dioxide (CO_2_) rather than organic ones [1,2,3,4,5,6,7,8,9]. Drugs such as antibiotics are chemical substances for which SCF technology involves appropriate formulation to increase the drug’s efficiency based specially on particle size, morphology and surface structure [2,10,11]. At present, the oldest and most frequently used antibiotics are Penicillins [12] which belong to the α-lactam group [13] because they have in their chemical structure an α-lactam ring fused to a thiazolidine ring [14]. Many years after their discovery by Fleming, penicillins are still the most effective treatment of diseases due to bacterial infections as Staphylococus and Syphilis [15,16,17]. Among them, the most important on the commercial plan are *Penicillin G* and *Penicillin V* [18].

Development of SCF processes for fine particle formation is important and is dependent on: “how much measurement of experimental drug’s solubility in SCFs is accurate” and its modeling to assure a suitable process design [19,20]. Solubility data in SCF knowledge and their variation under operating temperature and pressure are considered the first step to assess capabilities of SCF extraction and area criterion to choose a process among the different that exist. Thus, the phase-equilibrium and thermo-dynamical behavior allow to find optimal conditions (pressure and temperature) [21]. As an example, in the RESS process, it is crucial to have a pressure high enough and a temperature sufficiently below the melting point of the solid drug to warrant solubility in the SCF [14,22]. Many other processes exist and details can be found in the literature [23,24,25,26,27,28]. These processes to measure experimental solubility data of complex, polar and voluminous substances as Penicillins in supercritical CO_2_ is long and time-consuming, which explains the researchers’ interest in mathematical modeling. In their reviews, Brenneck and Eckert [29] and Johnston et al. [30] have discussed globally the analysis and modeling of this special phase equilibrium. To represent the thermodynamic behavior of these mixtures considered as largely asymmetric, models frequently applied are cEoSs, because they represent a fundamental tool [31,32]. However, disadvantages associated to this type of equation are many, even when solubility data are available because it is important(in the majority of cases) to insert other adjustable parameters [10,33,34]. The results of predicting solubility data in SC CO_2_ using cEoSs related to different mixing rules are affected by the estimation of some physical properties as acentric factors, critical constants and sublimation pressures of the solid drugs [35]. Here, the focus is on the sublimation pressure of *Penicillin G* and *Penicillin V*, which is the principal influencer on solubility and considered as an ad hoc adjustable parameter. To present this review, the steps aforementioned in the abstract are followed and detailed gradually.

## 2. Modeling and Thermodynamic Basis

In binary systems, the phase equilibrium state can be described by intensive variables as: the pressure *P*, the temperature *T* and mole fractions of the components ($y_{1},y_{2}$). Equilibrium is reached when chemical potentials of the i components ($\mu_{i}$) are equal at different phases according to Gibbs’ law [36]. Moreover, chemical potential’s equality is transformed into a fugacity ($f_{i}$) equality [37,38,39]. In case of a binary mixture consisting of an SCF phase and solid phase, the equality at equilibrium for the solute (2) is written as follows:

(1) $$f_{2}^{S}=f_{2}^{SF}$$

where $f_{2}$ denotes fugacity of solid solute in the solid phase (S) and in the supercritical fluid phase (SF), respectively. As the solid phase is considered to be pure, we have equality $f_{2}^{S}=f_{2}^{oS}$, where $f_{2}^{oS}$ is the fugacity of the pur solid solute and is given by Equation (2):

(2) $$f_{2}^{os}=P_{2}^{sub}\varphi_{2}^{sub}\exp\left[\int_{P_{2}^{sub}}^{P}\left(\frac{v_{2}^{s}}{RT}\right)dP\right]$$

where $P_{2}^{sub}$ is a solid solute’s sublimation pressure at system temperature, $v_{2}^{s}$ is its molar volume and $\varphi_{2}^{sub}$ is its fugacity coefficient at temperature *T* and at pressure $P_{2}^{sub}$ and is equal to 1. Integrating Equation (2) leads to:

(3) $$f_{2}^{os}=P_{2}^{sub}\exp\left[v_{2}^{s}\frac{P-P_{2}^{sub}}{RT}\right]$$

The fugacity of the solid solute in SCF phase is given by:

(4) $$f_{2}^{SF}=y_{2}\varphi_{2}^{SF}P$$

where $y_{2\;}$ is molar fraction of the solid solute in SCF phase and represents its solubility, since $\varphi_{2}^{SF}$ is its fugacity coefficient and is given by the thermodynamical expression below:

(5) $$\ln\varphi_{2}^{SF}=\frac{1}{RT}\int_{V}^{\infty }\left[{\left(\frac{\partial P}{\partial n_{2}}\right)}_{T,V,n_{1}}-\frac{RT}{V}\right]dV-\ln Z\;\;$$

where *R* is the gas constant, *V* is the total volume and *Z* is the compressibility factor $\left(Z=\frac{PV}{RT}\right)$.

Combining Equations (1), (3) and (4) gives:

(6) $$y_{2}=\left(\frac{P_{2}^{sub}}{P}\right)\frac{\exp\left(\frac{v_{2}^{s}}{RT}\left(P-P_{2}^{sub}\right)\right)}{\varphi_{2}^{SF}}$$

After considering that the operating pressure is much higher than $P_{2}^{sub}$, the above equation is reduced to Equation (7):

(7) $$y_{2}=\frac{P_{2}^{sub}}{P\varphi_{2}^{SF}}\exp\left(\frac{v_{2}^{S}P}{RT}\right)$$

The molar solubility $y_{2}$ is obtained by the compressed gas model given by Prausnitz et al. [36].

Penicillins’ properties $\left(v_{2}^{S},P_{2}^{sub}\right)$ and cEoS with specific mixing rules are required to calculate $y_{2\;}$ from Equation (7). The fugacity coefficient $\varphi_{2}^{SF}$ is the property obtained from thermodynamics-based model, which is differently to the solute’s properties which have to be estimated from other independent information.

This work is interested in the way adopted by Gordillo et al. [40] to estimate $P_{2}^{sub}$. Researchers considered it as a “second adjustable parameter” [41] to be calculated as $k_{12}$ by minimizing the absolute average relative deviations (AARD's) between experimental $\left(y_{2}^{ex}\right)$ and calculated $\left(y_{2}^{ca}\right)$ drug’s solubility, which is defined by Equation (8):

(8) $$AARD\left(\%\right)=\frac{100}{N}\overset{N}{\underset{i=1}{\sum }}\left(\frac{\left|y_{2}^{ex}-y_{2}^{ca}\right|}{y_{2}^{e}}\right)\;;\;N:\;number\;of\;data\;points$$

## 3. cEoSs Needed

Here are given the three cubic equations of states needed in the presentation of this work:

- Redlich-Kwong (RK cEoS):

(9) $$P=\frac{RT}{\left(v-b\right)}-\frac{a\alpha \left(T\right)}{v\left(v+b\right)}\;\;;\;\;\;\;\;\;\alpha \left(T\right)=\frac{1}{\sqrt{T}}$$

- Soave-Redlich-Kwong (SRK cEoS):
  (10) $$P=\frac{RT}{\left(v-b\right)}-\frac{a\;\alpha \left(T_{r},\omega \right)}{v\left(v+b\right)}\;;\;\alpha \left(T_{r},\omega \right)={\left[1+s\left(1-\sqrt{\;T_{r}}\right)\right]}^{2};\;T_{r}=\frac{T}{T_{c}};\;s=0.48+1.574\omega -0.176\omega^{2}$$
- Peng-Robinson (PR cEoS):
  (11) $$P=\frac{RT}{v-b}-\frac{a}{v\left(v+b\right)+b\left(v-b\right)}\left(ationsneeded\right)$$
  where ω is the acentric factor and $T_{c}$ and $T_{r}$ are the critical and reduced temperatures, respectively. The conventional mixing rules of van der Waals and combination mixing rules of Lorentz-Berthelot are given below [40]:
  (12) $$a=\underset{i}{\sum }\underset{j}{\sum }y_{i}y_{j}a_{ij}\;;\;\;\;b=\underset{i}{\sum }y_{i}b_{i}$$
  (13) $$\{\begin{array}{c} a_{ij}=0.42748\frac{R^{2}T_{C_{ij}}^{2,5}}{P_{C_{ij}}}\;;\;T_{C_{ij}}=\left(1-k_{ij}\right)\sqrt{T_{C_{i}}T_{C_{j}}}\\ P_{C_{ij}}=\frac{Z_{C_{ij}}RT_{C_{ij}}}{V_{C_{ij}}};\;Z_{C_{ij}}=\frac{Z_{C_{i}}+Z_{C_{j}}}{2}\;;\;V_{C_{ij}}={\left(\frac{V_{C_{i}}^{1/3}+V_{C_{j}}^{1/3}}{2}\right)}^{2} \end{array}$$

## 4. Review and Discussion of Literature Results

From the aforementioned equations, it is clear that there is one direct regressing parameter ($k_{ij}$), and that properties of solutes (Penicillins) are needed to estimate the solubility $y_{2}$.

### 4.1. Penicillin G

For *Penicillin G*, Gordillo et al. [40] used two equations of states Redlich-Kwong (RK cEoS) [42] and Soave-Redlich-Kwong (SRK cEoS) [43] with the Lorentz-Berthelot combination mixing rule given by Equation (9). They have measured molar volume $v_{2}^{S}$ experimentally and obtained$\;0.2261\;lmol^{-1}$ as value, and used different group contribution methods *GCM* to estimate acentric factor and critical coordinates, and considered $P_{2}^{sub}$ as a “second adjustable” parameter.

Gordillo et al. used the experimental solubility data of *Penicillin G* in SC CO_2_, to obtain an estimated $P_{2}^{sub}$ by regression with RK cEoS and SRK cEoS. Table 1 presents their results.

In systems involving SCFs, a model’s ability and success are evaluated by the (*AARD*) approach [44], since it is the assessment most widely used [39,45]. Table 1 shows an *AARD* of 21% and 23%, which reflect the globally an acceptable correlation results of RK and SRK cEoSs, respectively. However, quantitative analysis of the error percentage indicates considerable difference between different obtained values of $P_{2}^{sub}$.

### 4.2. Penicillin V

For *Penicillin V*, Ko et al. [46] have used the equation of state of Peng-Robinson (PR cEoS) given by Equation (7) and conventional mixing rules of van der Waals. For the molar volume, they used a value of $213\;cm^{3}/mole$ estimated by a group contribution method. However, later in 1993, Vafai and co-workers [47] calculated the molar volume experimentally and published a value of $243.2\;cm^{3}/mole$, which entails a 12% error on the molar volume value. For the acentric factor and critical constants, they used group contribution methods GCM. For sublimation pressure, they used two values (Table 2): the first value is estimated by a modified Clausius-Clapeyron equation and the second value is obtained by regressing experimental solubilities by PR cEoS, as done by Gordillo et al. The regression results for *Penicillin V* data obtained by Ko et al. are displayed in Table 2. The deviations obtained are generally large but very close (same order of magnitude) for the two cases although the sublimation pressures are different from one case to another. Their difference is presented as an error percentage, and as we can see thisis considerably large.

For the two binary systems (*Penicillin G*–CO_2_) and (*Penicillin V*–CO_2_), deviations of *AARD* are relatively large and very pointed at high temperatures (333.15 K and 334.85 K). However, the question is what is the reliable value of sublimation pressure as long as the deviations *AARD* are close? 

To address this question, an attempt based on the use of the modified form of PR cEoS [48,49] is presented.

## 5. Schmitt and Reid Modified PR cEoS

Using cubic equations of states in a “traditional manner” requires a solute’s critical properties and acentric factor, which are generally unavailable for large chemicals with complex structures such as *Penicillins* [50].

The problem gets complicated when some methods applied to small molecules are used for their estimation which introduces considerable additional errors in many cases and affects considerably the capabilities of the considered cEoS because uncertainty in the calculation contributes considerably to the inaccurate use of the cEoS, since estimated values are regarded as pseudo-properties [31,37,51].

To estimate the critical properties of *Penicillin G*, Gordillo et al. [10] used different group contribution methods (GCM) together with different cEoSs and mixing rules. They obtained different values from each GCM and they asserted in their conclusion that “the choice of GCM is more important than the choice of the cEoS itself”. From this point, cEoSs cannot be blamed for inaccurate results [52].

Peng-Robinson cEoS is considered by researchers as the most well-known and widely used due to its flexibility and simplicity [53,54,55]. Schmitt and Reid presented a modification to this equation [48,56] which makes it possible to avoid estimation of a solute’s critical properties. 

In fact, they excluded the binary interaction parameter, supposed that solute’s parameters $a_{2}$ and $b_{2}$ are independent of temperature and considered them as adjustable parameters. Additionally, terms containing $y_{2}$ in the combining and mixing rules were eliminated due to their small values. 

They proposed the simplified form for the fugacity coefficient given by Equation (14) below [48]:

(14) $$\begin{array}{ll} \ln\varphi_{2}^{SF}= & \left(\frac{b_{2}}{b_{1}}\right)\left(Z-1\right)-\ln\left[\frac{P\left(V-b_{1}\right)}{RT}\right]\\ & -\left(a_{1}/8^{1/2}RTb_{1}\right)\left(2\sqrt{\frac{a_{2}}{a_{1}}}-\left(\frac{b_{2}}{b_{1}}\right)\right)\times \ln\left[\frac{\left(V+2.414b_{1}\right)}{\left(V-\sqrt{2}b_{1}\right)}\right] \end{array}$$

Parameters $a_{1}$ and $b_{1}$ are calculated according to van der Waals’s classical mixing rules using pure carbon dioxide properties [36,48]:

(15) $$\{\begin{array}{c} a_{1}=\frac{0.4572R^{2}T_{C_{1}}^{2}}{P_{c_{1}}}\times {\left[1+\left(0.3746+1.5423\omega_{1}-0.2699\omega_{1}^{2}\right)\left(1-\sqrt{T_{r}}\right)\right]}^{2}\\ \;b_{1}=\frac{0.07780RT_{c_{1}}}{P_{c_{1}}} \end{array}$$

Using Equations (14) and (15) together with $P_{2}^{sub}$ obtained in Section 4.1 and Section 4.2, the molar solubility of *Penicillin G* and *Penicillin V* can be calculated by Equation (7).

- Regression of the experimental data (N = 18) for *Penicillin G* with modified PR cEoS is done by the implementation of $P_{2}^{sub}$, one of whichis obtained by SRK cEoS ($P_{2}^{sub}$-SRK) and the other is obtained by RK cEoS($P_{2}^{sub}$-RK). The results are presented in Table 3.

From Table 3, we can see that the use of sublimation pressure ($P_{2}^{sub}$-SRK) leads to very large deviations, both globally as well as for specific temperature values.

The results are better visualized by representations; here, plots are presented as molar solubility versus density instead of pressure because they are more informative [57].

**Figure 1:**
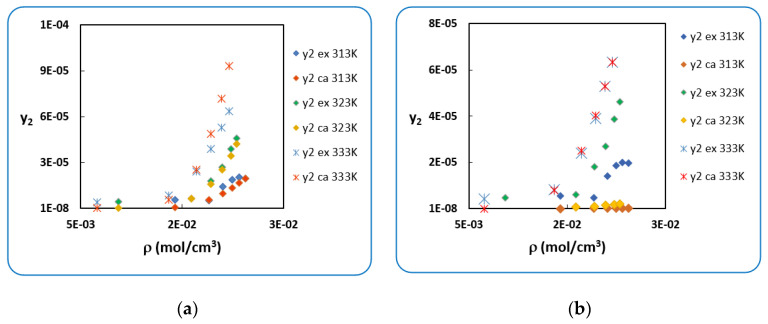
Experimental and calculated solubility of *Penicillin G* versus density. (**a**) ($P_{2}^{sub}$-RK). (**b**) ($P_{2}^{sub}$-SRK).

In Figure 1 below, in (a) and (b), the calculated solubilities of *Penicillin G* by modified PR cEoS using $P_{2}^{sub}$ estimated by SRK cEoS are not in agreement with the experimental ones and show considerable deviations.

**Figure 2:**
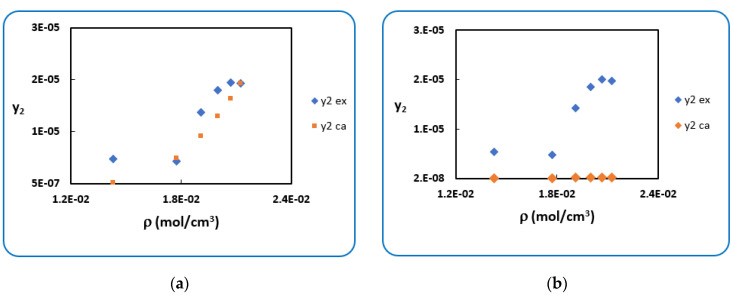
Experimental and calculated solubility of *Penicillin G* at T = 313K: (**a**) ($P_{2}^{sub}$-RK). (**b**) ($P_{2}^{sub}$-SRK).

Figure 2 shows the large difference obtained at 313K, in (a) all calculated solubilities with modified PR cEoS and experimental ones which are in concordance when using the ($P_{2}^{sub}$-RK), which is not the case in (b) when using($P_{2}^{sub}$-SRK).

- The same is true for *Penicillin V*; regression of the solubility data (N = 24) with modified PR cEoS is done by implementing$\;P_{2}^{sub}$ given in Table 2 and obtained results are displayed in Table 4.

**Figure 3:**
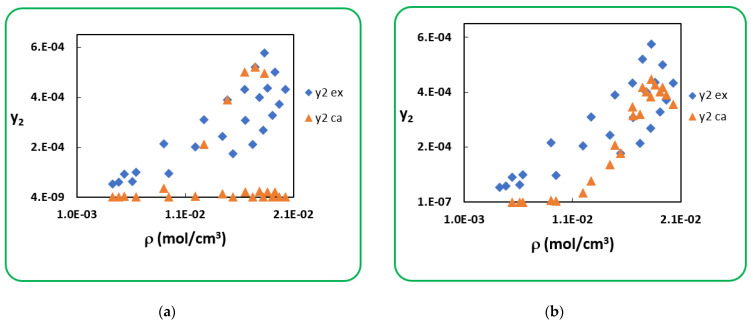
Experimental and calculated solubility of Penicillin V (global representation): (**a**) ($P_{2}^{sub}$-PR), (**b**) ($P_{2}^{sub}$-Clapeyron).

**Figure 4:**
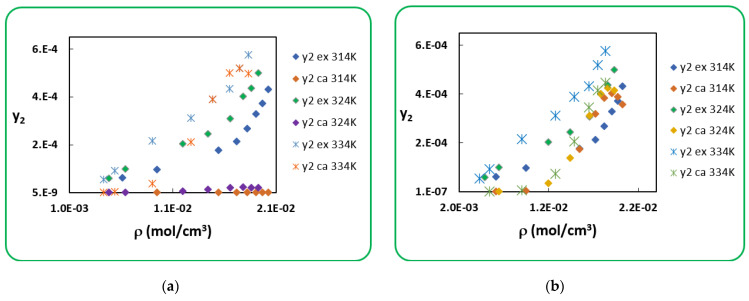
Experimental and calculated solubility of *Penicillin V* (temperature-by-temperature representation): (**a**) ($P_{2}^{sub}$-PR), (**b**) ($P_{2}^{sub}$-Clapeyron).

The use of sublimation pressure ($P_{2}^{sub}$-PR) leads to larger deviations than the use of sublimation pressure ($P_{2}^{sub}$-Clapeyron). The results are represented in figures below; Figure 3 clearly shows that globally, the concordance between the experimental and calculated solubility is inexistent in (a) and that this is less marked in (b). Figure 4 shows the same conclusion in a temperature-by-temperature representation.

The results of this section can address the question posed before; thus, for Gordillo et al., ($P_{2}^{sub}$-RK) is more accurate than ($P_{2}^{sub}$-SRK) and for Ko et al., ($P_{2}^{sub}$-Clapeyron) is more accurate than ($P_{2}^{sub}$-PR). 

**Figure 5:**
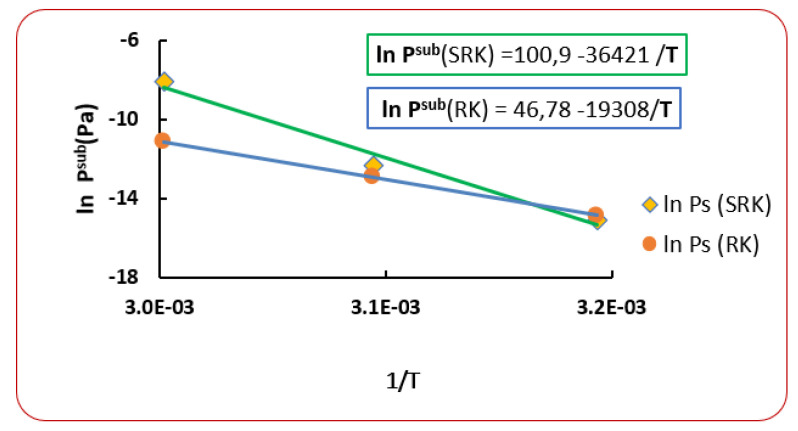
Interpolation of the sublimation pressures obtained for *Penicillin G* by the (Clausius-Clapeyron) equation.

From another point of view, when fitting the obtained sublimation pressures to the equation of Clausius-Clapeyron (Equation (17)), very high values for sublimation enthalpy are obtained. For example, in Figure 5 are presented the results for *Penicillin G*, and from the slopes of the two straight lines, a sublimation enthalpy of 302.8 kJ/moland 160.5 kJ/mol are found. These values are considered very high for a molecule such as *Penicillin*, which is not very complex and larger than others. These results lead to propose a new sublimation pressure.

## 6. Use of New Sublimation Pressure

Similar to many other drugs, Penicillins are polar, thermolabile and nonvolatile compounds. Because of that, they can decompose before their boiling point $T_{b}$ [53] and sublime before their melting point $T_{m}$ [58]. In both cases, their boiling temperature cannot be found [58], since some methods for sublimation pressure estimation involving these two specific temperatures (such as that of Mackay et al. [59] for example) can be avoided by considering other approaches, because the obtained results will represent vapor pressures instead of sublimation pressures [60].

For this purpose, another attempt by involving a new approach for the sublimation pressure is presented here. Recently, Nasri [61] have presented a different way to estimate $P_{2}^{sub}$, which is based on obtaining the two parameters $A^{S}$ and $B^{S}$ of the Clausius-Clapeyron equation given by Equation (12) below [62]. This requires three steps: first, checking the consistency of the solubility data; second, correlating the data by the model of Mendez-Santiago-Teja [63] to have $A^{S}$; third, correlating the data by the Bartle’s model [64] to obtain parameter $B^{S}$, which represents the sublimation enthalpy $\Delta H^{s}$ [65,66,67,68,69,70].

**Figure 6:**
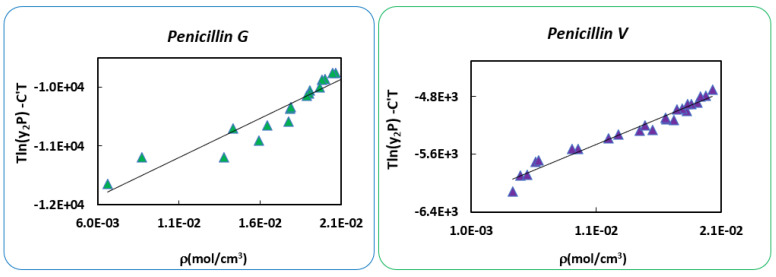
Consistency of the solubility data.

From Figure 6, it can be seen clearly that the solubility data of the two Penicillins generally follow a linear trend. For *Penicillin G*, we have two points that are far from the line, these points are (323.15 K; 100 bar) and (333.15 K; 100 bar) and are eliminated in the correlation’s step to be able to estimate accurate parameters, and thus, an accurate $P_{2}^{sub}$, and are reconsidered in the calculation step of molar solubility with modified PR cEoS.

Table 5 gives the results of the *Penicillins* data’s correlation, which is very acceptable since the *AARD* is low. These results are used to estimate the new sublimation pressure [62] according to Equation (16).

(16) $$\ln p_{2}^{Sub}=A^{S}-\frac{B^{S}}{RT}$$

**Figure 7:**
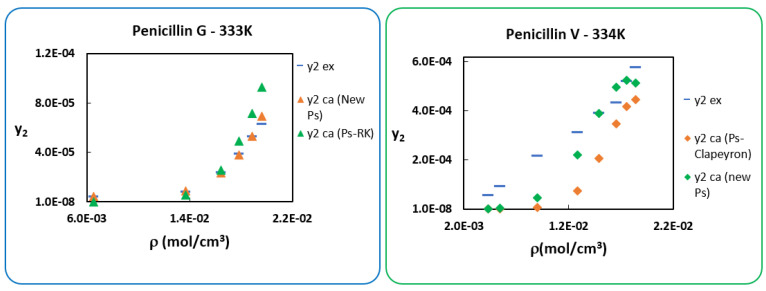
Comparison at two temperatures between the experimental and calculated solubilities.

As in Section 5, the new sublimation pressures in Table 5 are used to calculate the molar solubility $y_{2}$ of the two *Penicillins* with the modified PR cEoS. The results are displayed in Table 6 and compared to those of Section 4. It is very clear to see that the *AARDs* have decreased considerably for the two *Penicillins* and even more for *Penicillin G* with an *AARD* of only 4% at 333 K. For better visualization, the new solubilities calculated at higher temperatures together with experimental ones, as well as those considered the best in the previous section, are presented in Figure 7. Very good agreement between the obtained solubilities involving the new $P_{2}^{sub}$ and experimental ones for both penicillins is observed.

## 7. Conclusions

The sublimation pressure is very important in the thermodynamic modeling of a drug’s solubility in SC CO_2_ using the cubic equations of states approach. This work focused on this predominant thermophysical property.

Many researchers have considered it as an adjustable parameter; here, we present a review, address the study’s research question and use a new approach for sublimation pressure to considerably improve the results obtained from the use of the modified Peng-Robinson cEoS, in which the effect of the estimated critical coordinates of the solid solutes (Penicillins) is highly reduced. The results obtained for both *Penicillin G* and *Penicillin V* are very promising, since the *AARDs* have decreased considerably (just 4% in some cases).

Moreover, it should be noted that considering the sublimation pressure as an adjustable parameter will further complicate the calculations; additionally, cEoSs still have many advantages, even when the supercritical fluid phase is considered.

## Figures and Tables

**Table 1:** $P_{2}^{sub}$ regressed by RK and SRK EoS for *Penicillin G*-CO_2_ and error percentage.

*T* (K)	$P_{2}^{sub}\;(\mathrm{Bar})$ RK [40]	*AARD* (%) [40]	$P_{2}^{sub}\;(\mathrm{Bar})$ SRK [40]	*AARD* (%) [40]	$\mathrm{Error}\left[\frac{\left|\left(P_{2}^{sub}-SRK\right)-\left(P_{2}^{sub}-RK\right)\right|}{\left(P_{2}^{sub}-SRK\right)}\right]$
313.15	$3.55\times 10^{-12}$	23	$2.82\times 10^{-12}$	21	26%
323.15	$2.24\times 10^{-11}$	23	$4.57\times 10^{-11}$	21	51%
333.15	$1.44\times 10^{-10}$	23	$3.09\times 10^{-9}$	21	95%

**Table 2:** $P_{2}^{sub}$ estimated by two equations for *Penicillin V*-CO_2_ and error percentage.

*T*(K)	$P_{2}^{sub}(\mathrm{Bar})$ Clausius [46]	AARD (%)	$P_{2}^{sub\;}(\mathrm{Bar})$ PR [46]	AARD (%)	$\mathrm{Error}\left[\frac{\left|\left(P_{2}^{sub}-PR\right)-\left(P_{2}^{sub}-Clausius\right)\right|}{\left(P_{2}^{sub}-PR\right)}\right]$
314.85	$5.53\times 10^{-10}$	37.85	$1.15\times 10^{-10}$	36.23	381%
324.85	$1.54\times 10^{-9}$	42.46	$9.10\times 10^{-9}$	40.25	83%
334.85	$3.83\times 10^{-9}$	54.38	$3.93\times 10^{-7}$	41.30	99%

**Table 3:** Regression results with modified PR EoS for *Penicillin G.*

	$\mathrm{Using}\;(P_{2}^{sub}-\mathrm{SRK})$	$\mathrm{Using}\;(P_{2}^{sub}-\mathrm{RK})$
$a_{2}\left(Pa{\left(m^{3}/mol\right)}^{2}\right)$	1.63 × 10^−4^	2.19 × 10^−4^
$b_{2}\left(m^{3}/mol\right)$	1.89 × 10^−4^	1.98 × 10^−4^
(*AARD* %) global	70.86	27.98
(*AARD*%) for 313 K	98.58	28.61
(*AARD*%) for 323 K	95.04	23.10
(*AARD*%) for 333 K	18.95	41.51

**Table 4:** Regression results with modified PR cEoS for *Penicillin V.*

	$\mathrm{Using}\;(P_{2}^{sub}-\mathrm{PR})$	$\mathrm{Using}\;(P_{2}^{sub}-\mathrm{Clapeyron})$
$a_{2}\left(Pa{\left(m^{3}/mol\right)}^{2}\right)$	1.87 × 10^−4^	2.80 × 10^−4^
$b_{2}\left(m^{3}/mol\right)$	2.72 × 10^−4^	2.93 × 10^−4^
(*AARD* %) global	79.5	48.5
(*AARD*%) for 314.85 K	99.5	41.7
(*AARD*%) for 324.85 K	100.1	43.4
(*AARD*%) for 334.85 K	48.1	60.3

**Table 5:** Results of the correlating data with the two empirical models and the newsublimation pressures obtained.

	Mendez-Santiago-Teja Model			Bartle’s Model	
	*Penicillin G*	*Penicillin V*		*Penicillin G*	*Penicillin V*
A’=	−11,475.4	−5495.9	a =	25.1	12.6
B’=	165,852.2	73,223.7	b =	−10,260.3	−5049.7
C’=	26.2	12.8	c =	1.2 × 10^−2^	5.05 × 10^−3^
*AARD*% =	24.6	17.01	*AARD*% =	16.9	16.1
			$\Delta H^{s}$_estimated_ (kJ/mol)	85.3	41.9
$\ln P_{2}^{sub}\left(Pa\right)=C^{\prime }-\frac{\Delta H^{S}}{RT}$					
*Penicillin G*: $P_{2}^{sub}=e^{\left(26.2-\frac{85.3}{RT}\right)}$			*Penicillin V*: $P_{2}^{sub}=e^{\left(12.8-\frac{41.9}{RT}\right)}$		

**Table 6:** Results of the calculated solubilities using new sublimation pressures.

	*AARD*% (*Penicillin G*)			*AARD*% (*Penicillin V*)	
	$\mathrm{New}\;P_{2}^{sub}$	$P_{2}^{sub}-\mathrm{RK}$		$\mathrm{New}\;P_{2}^{sub}$	$P_{2}^{sub}-\mathrm{Clapeyron}$
*T* = 313.15 K	27.4	28.6	*T* = 314.85 K	26.7	41.7
*T* = 323.15 K	11.1	23.1	*T* = 324.85 K	26.4	43.4
*T* = 333.15 K	4.0	41.5	*T* = 334.85 K	40.6	60.3
